# Multi-Level Attribute-Guided-Based Adaptive Multi-Dilated Convolutional Network for Image Aesthetic Assessment

**DOI:** 10.3390/jimaging11120420

**Published:** 2025-11-21

**Authors:** Sumei Li, Mingxuan Xie, Wei Xiang

**Affiliations:** 1 School of Electrical and Information Engineering, Tianjin University, Tianjin 300072, China; lisumei@tju.edu.cn; 2 School of Computing, Engineering and Mathematical Sciences, La Trobe University, Melbourne, VIC 3086, Australia; w.xiang@latrobe.edu.au; 3 College of Science and Engineering, James Cook University, Cairns, QLD 4878, Australia

**Keywords:** image aesthetics assessment, aesthetic attributes, attention mechanism, dilated convolution

## Abstract

Image aesthetic assessment (IAA) is crucial for both scientific research and practical applications, and numerous studies have achieved promising performance. However, they still exhibit two major limitations: the neglect of hierarchical interactions between attribute features and aesthetic features, and the distortion of the original aspect ratio during image preprocessing, which leads to a loss of aesthetic information. To address these issues, we propose a Multi-level Attribute-Guided Adaptive Multi-Dilated Convolutional Network (MAADN), which leverages multi-level attribute features to guide aesthetic assessment and reduces the negative impact of image preprocessing through adaptive dilated convolution. Specifically, we employ a dual-branch architecture: one branch extracts multi-level attribute features, while the other learns aesthetic features under the guidance of these attributes. We further design an Attention-based Attribute-Guided Aesthetic Module (AGAM), which utilizes visual attention mechanisms to enhance the guidance of attributes. Additionally, we design an Adaptive Multi-Dilate Rate Convolution Module (AMDM) that generates weights adaptively through the network to fuse dilated convolution features with different dilation rates, rather than simply calculating weights based on aspect ratios. This approach effectively alleviates the negative effects of image preprocessing while maintaining training flexibility. Extensive experimental results demonstrate that the proposed model outperforms current state-of-the-art approaches. Furthermore, visual analysis confirms MAADN’s precise localization capability for aesthetically critical regions.

## 1. Introduction

With the rapid development of the internet and social media, images have become a crucial medium for information exchange, driving an increasing demand for high aesthetic quality. Image aesthetics assessment (IAA) is a computational task for evaluating the visual appeal of an image, which plays an important role in applications such as image enhancement [1], photographic composition [2], album management [3], and photo recommendation [4], and is now receiving more and more attention from both academia and industry.

Existing IAA methods are broadly categorized into two groups: hand-crafted feature-based IAA and deep learning-based IAA. Early studies [5,6,7] mainly utilized handcrafted features designed by photographic rules or perceptual differences to binary classify images for aesthetic quality. With the development of deep learning, IAA models based on convolutional neural networks (CNNs) have demonstrated their advantages and gradually become the mainstream method [8,9,10]. Lu et al. [11] were the first to utilize a deep learning approach to learn aesthetic scores from images, and Talebi et al. [12] proposed a model for predicting aesthetic quality distribution using Earth Mover’s Distance (EMD) loss. However, these methods only extracted features from the image itself and ignored the important role of image attributes. Research shows that human judgment of image aesthetic is inseparable from their perception of various visual attributes [13]. Therefore, many methods began to utilize image attributes to assist IAA tasks. Some studies used pre-trained neural networks to extract image attribute features to assist IAA [14,15]. Other studies constructed multi-task learning frameworks to jointly optimize attribute recognition and aesthetic evaluation [16,17,18]. However, these methods typically fused the attribute and aesthetic features only from the deepest layer of the network to predict the aesthetic quality, ignoring the hierarchical interaction relationship between attribute features and aesthetic features. Research shows that people’s evaluation of image aesthetic quality is a progressive process from low-level features to high-level semantics [19,20]. Inspired by this, we propose a Multi-level Attribute-Guided-based Adaptive Multi-Dilated Convolutional Network (MAADN). This network achieves multiple guidance fusions of attribute and aesthetic features extracted from different levels. Furthermore, we design an Attention-based Attribute-Guided Aesthetic Module (AGAM) within MAADN to effectively facilitate the guidance of aesthetic features by attribute features.

In addition, CNN-based IAA models are limited by fixed-size inputs, typically requiring images to be resized or cropped to the same dimensions. However, such preprocessing methods disrupt the spatial composition and structural integrity of images. This leads to the loss of aesthetic information. Consequently, the correspondence between the aesthetic quality of the processed image and its original label becomes less accurate. Therefore, how to design input tailored for the IAA algorithm has become a key research direction. Mai et al. [21] introduced an adaptive spatial pooling layer, which pooled visual features from images of any size into fixed-size features, enabling the network to adapt to inputs of any image size. However, the adaptive pooling layer blurred a significant amount of features during computation. Later, Chen et al. [22] proposed an IAA model that calculated the weights of different dilated convolutions according to the aspect ratio of the input image, retaining the input image composition. However, this design not only required the use of small batch sizes, which limited training flexibility, but also resulted in relatively low performance, achieving only 0.649 SRCC and 0.671 PLCC on the AVA dataset. Therefore, we design an Adaptive Multi-Dilate Rate Convolution Module (AMDM) in which its weights are adaptively learned from input images. And it does not require the use of small batch data during training. This method not only reduces the damage caused by pre-processing to the aesthetic features of the image, but also eliminates the inflexibility of using small batch data during the model training process. Consequently, it achieves 0.714 SRCC and 0.728 PLCC on the AVA dataset.

The contributions of this work can be summarized with the following points:

- We propose a new IAA model, named Multi-level Attribute-Guided-based Adaptive Multi-Dilated Convolutional Network (MAADN), which first implements multi-level guidance from attribute features to the IAA task, simulating the hierarchical mechanism of the human visual system. Meanwhile, this model can achieve a better consistency with subjective aesthetic quality ratings.
- We design an Attention-based Attribute-Guided Aesthetic Module (AGAM), which effectively implements the guidance of attribute features on aesthetic features through the attention mechanism, improving the accuracy and interpretability of the model.
- We design an Adaptive Multi-Dilate Rate Convolution Module (AMDM) that dynamically weights features from parallel dilated convolutions with different dilation rates. This effectively alleviates the negative impact of image preprocessing and the constraint of small-batch training.

The rest of the paper is structured as follows. In Section 2, a brief review of existing IAA models is presented. Section 3 describes the details of the proposed model, MAADN. Section 4 gives the experimental results and analysis. Finally, Section 5 summarizes the paper.

## 2. Related Works

In this section, we briefly review existing IAA models. We categorize them into two types: hand-crafted feature-based IAA and deep learning-based IAA.

### 2.1. Hand-Crafted Feature-Based IAA

Early research was mainly based on hand-crafted feature-based IAA, and its main process is as follows: First, features, which can reflect the aesthetics of an image, are designed according to the rules of photography, color theory, and other aesthetic knowledge. These features are then fed into machine learning models, such as Support Vector Machine (SVM) [23] or Bayesian Classifier [24], to complete the IAA task. For example, Datta et al. [6] proposed 56 features (including color, saturation, etc.) for measuring image quality to distinguish between aesthetically pleasing or unattractive images. Nishiyama et al. [25] extracted the local color descriptors and then constructed histograms as the features for aesthetically pleasing image classification. Later, some studies utilized generic image descriptors to measure image aesthetics, such as Bag of Visual Words (BOV) [26] and Fisher Vector (FV) [27]. Although these hand-crafted features had clear physical meanings, they only characterized a limited understanding of aesthetics due to the highly abstract nature of image aesthetics. Their representational power was generally weak and insufficient for the IAA task.

### 2.2. Deep Learning-Based IAA

With the significant advances in deep learning, the focus of research has shifted to deep learning-based IAA. In 2014, Lu et al. [11] introduced CNNs to the IAA task for the first time, sparking the development of diverse methods. We categorize these subsequent methods into “Attribute-guided Methods” and “Composition-preserving Methods” based on their technical approaches, and briefly review other related IAA methods.

#### 2.2.1. Attribute-Guided Methods

Numerous methods have utilized image attributes to assist in IAA. Li et al. [15] employed a pre-trained feature extraction network to extract image attribute features and thematic features. Subsequently, they utilized graph convolutions to uncover intrinsic correlations between visual attributes and image themes, ultimately generating aesthetic prediction results. Kao et al. [16] designed a multi-task learning network aimed at jointly learning the association between semantic attributes of subjects and aesthetic quality. Pan et al. [18] proposed an image aesthetic evaluation method that leverages aesthetic attributes as privileged information through adversarial learning to enhance the accuracy of aesthetic score predictions. Shu et al. [28] proposed an image aesthetic evaluation method based on privileged multi-task learning, which jointly models the multiple dependencies between attributes and aesthetics by incorporating ranking, similarity, prior probability, and adversarial loss. Although these methods made significant progress, they did not thoroughly consider the hierarchical guidance of aesthetic attributes, which aligns with human aesthetic experience. To address this limitation and inspired by the hierarchical structure of the human visual system, we propose a Multi-level Attribute-Guided-based Adaptive Multi-Dilated Convolutional Network (MAADN), which implements multiple guidance for aesthetic features through these extracted attribute features.

#### 2.2.2. Composition-Preserving Methods

Since CNNs are limited by fixed-size inputs, how to handle images of varying sizes has become an urgent issue to address. Researchers first considered cropping images into multiple patches of fixed size to accommodate CNNs’ constraints on input size. Lu et al. [29] randomly cropped multiple patches from an image for prediction and fused the features of different patches based on statistics such as maximum, minimum, and mean. However, cropping damages the overall aesthetic appeal of the image and reduces the performance of the IAA model. Later, many studies attempted to solve the size limitation of input images in CNNs. Mai et al. [21] proposed an adaptive spatial pooling operation, which was added in front of the regular convolutional and pooling layers to directly process the original image without scaling. Jin et al. [30] constructed an aesthetic adaptive module that could adapt to any size of the input image, and filled the input image to a uniform size and fed it to the aesthetic adaptive module to extract features. Chen et al. [22] proposed an IAA model based on adaptive fractional dilated convolution, which could keep the aspect ratio of the original image unchanged. Based on this, we design an Adaptive Multi-Dilate Rate Convolution Module (AMDM), which simulates the adaptive perception characteristics of the human brain on images with different aspect ratios by dynamically weighting features obtained from convolution with different dilation rates, thereby reducing the damage to image aesthetic quality caused by image preprocessing, and without relying on small batch data during training.

#### 2.2.3. Other Related IAA Methods

Many recent studies have used Transformer and multimodal models to assess the aesthetic quality of images. Wang et al. [31] proposed a transformer-based model incorporating Regional Patch Attention to compute aesthetic weights for different image regions, enabling simultaneous aesthetic evaluation and cropping with enhanced global feature modeling. Li et al. [32] designed an attribute-assisted multimodal memory network to enhance aesthetic representation by capturing perceptual information related to images and reviews through a memory network and refining the semantics of attributes shared by the two modalities through jump connections. Qi et al. [33] proposed a multimodal full transformer that integrates visual and textual streams via cross-attention fusion, unifying aesthetic classification, regression, and distribution prediction tasks while outperforming state-of-the-art methods. Wang et al. [34] proposed a framework leveraging Multi-modal Large Language Models (MLLMs) with Aesthetic Attribute Assessment and Scene-aware In-context Learning, enhancing interpretable image aesthetics assessment and achieving improved performance across multiple datasets. Although multimodal models often achieved better results than unimodal models, their application scope was somewhat limited because the required textual comment information was not always included in datasets or was difficult to obtain. Based on this, this paper focuses on the unimodal IAA approach.

Beyond the conventional scope of IAA, several recent studies have expanded its application. Li et al. [35] proposed a framework for evaluating the aesthetic quality of generated images and their alignment with text in sentiment and aesthetics, and utilized it to filter high-quality images for enhancing generative models. Wan et al. [36] proposed a Big Five personality trait-based aesthetic assessment model and a personality encoder to drive text-to-image models for generating personalized images that align with individual aesthetic preferences, achieving personalized short-text-to-image generation. Xiao et al. [37] proposed an aesthetic-oriented multi-granularity fusion network, introducing image aesthetic assessment into joint multimodal aspect-based sentiment analysis for the first time to improve sentiment recognition performance. Maerten et al. [38] constructed the first personalized Image Aesthetic Assessment(PIAA) dataset for artistic images, featuring rich image and personal attributes, and experimentally validated the performance and challenges of existing PIAA models on this dataset. Collectively, these works significantly expand the conventional boundaries of IAA, laying a solid foundation for its application in cutting-edge fields like generative AI and multimodal understanding, thereby collectively advancing the entire research domain.

## 3. Proposed Method

**Figure 1:**
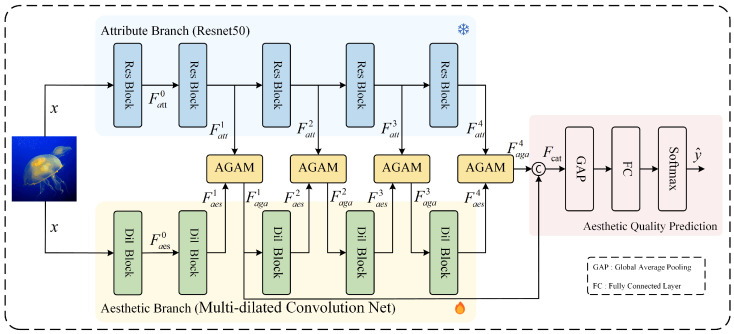
Architecture of the proposed Multi-level Attribute-Guided-based Adaptive Multi-Dilated Convolutional Network (MAADN).

In this section, we briefly introduce the proposed Multi-level Attribute-Guided-based Adaptive Multi-Dilated Convolutional Network (MAADN), with the overall structure shown in Figure 1. MAADN consists of four main parts: Attribute Branch, Aesthetic Branch, AGAM, and Aesthetic Quality Prediction. The Attribute Branch extracts multi-level attribute features, which then guide the aesthetic features extracted from the Aesthetic Branch through AGAM. The Aesthetic Branch includes the designed AMDM, which can reduce damage to image quality caused by the image preprocessing stage.

### 3.1. Overall Structure

**Figure 2:**
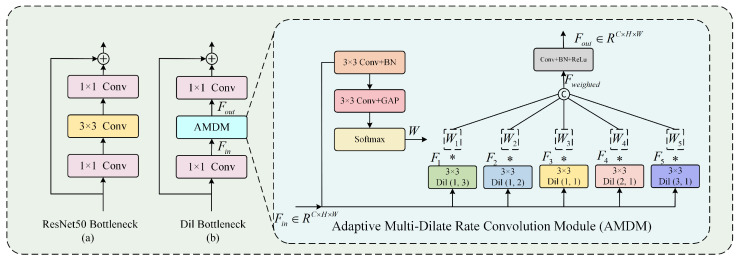
Architecture of the proposed Adaptive Multi-Dilate Rate Convolution Module (AMDM). The ∗ denotes multiplication.

Below, we detail the overall structure of the proposed network. First, for the Attribute Branch, we choose ResNet50 [39], which consists of five Res Blocks, as the backbone and pre-train it on the AADB dataset [40] by 11 visual attributes. The visual attributes include balancing elements, color harmony, content, depth of field, light, motion blur, object, repetition, rule of thirds, symmetry, and vivid color. After pre-training is complete, the weights of the network will be frozen. In the Aesthetic Branch, a Multi-Dilated Convolutional Network was constructed, consisting of five dilated convolution blocks named Dil Blocks. The Dil Block consists of a series of Dil Bottlenecks, which contain our designed AMDM. The structure of AMDM is shown in Figure 2 and will be introduced in Section 3.3. The Aesthetic branch does not require pre-training. Next, we will introduce our multi-level feature extraction process. We define the following notation for clarity and consistency:

- $F_{att}^{l}$: Feature map output from the *l*-th Res Block in the Attribute Branch
- $F_{aes}^{l}$: Feature map output from the *l*-th Dil Block in the Aesthetic Branch
- $F_{aga}^{l}$: Feature map output from the *l*-th AGAM module
- $\Psi_{att}^{l}\left(\cdot \right)$: Nonlinear transformation function of the *l*-th Res Block
- $\Psi_{aes}^{l}\left(\cdot \right)$: Nonlinear transformation function of the *l*-th Dil Block
- $\Psi_{AGAM}^{l}\left(\cdot \right)$: Nonlinear transformation function of the *l*-th AGAM

The feature extraction process at each level is defined as follows:

(1) $$\begin{array}{ccc} F_{att}^{l} & =\Psi_{att}^{l}\left(x\right), & \;\;\;\;\;\;\;\;\;l=0 \end{array}$$

(2) $$\begin{array}{ccc} F_{att}^{l} & =\Psi_{att}^{l}\left(F_{att}^{l-1}\right), & \;\;\;\;\;l\in \{1,2,3,4\} \end{array}$$

(3) $$\begin{array}{ccc} F_{aes}^{l} & =\Psi_{aes}^{l}\left(x\right), & \;\;\;\;\;\;\;\;l=0 \end{array}$$

(4) $$\begin{array}{ccc} F_{aes}^{l} & =\Psi_{aes}^{l}\left(F_{aes}^{l-1}\right), & \;\;\;\;\;\;\;\;\;l=1 \end{array}$$

(5) $$\begin{array}{ccc} F_{aes}^{l} & =\Psi_{aes}^{l}\left(F_{aga}^{l-1}\right), & \;\;\;\;\;l\in \{2,3,4\} \end{array}$$

(6) $$\begin{array}{ccc} F_{aga}^{l} & =\Psi_{AGAM}^{l}\left(F_{att}^{l},F_{aes}^{l}\right), & \;\;l\in \{1,2,3,4\} \end{array}$$

where *x* denotes the input image.

After that, we concatenate $F_{aga}^{1}$ and $F_{aga}^{4}$ to obtain $F_{cat}$, after which we predict the aesthetic distribution by a global average pooling layer (GAP), and finally by employing a fully connected layer (FC) and a softmax activation function. The final prediction process is computed as follows:

(7) $$F_{cat}=\mathrm{Concat}\left(F_{aga}^{1},F_{aga}^{4}\right)$$

(8) $$\hat{y}=\mathrm{Softmax}\left(\mathrm{FC}\left(\mathrm{GAP}\left(F_{cat}\right)\right)\right)$$

where Concat(·) denotes the channel-wise concatenation, GAP(·) denotes the global average pooling, and FC(·) denotes the fully connected layer. $\hat{y}$ represents the predicted aesthetic score distribution.

Our model is optimized by minimizing the Earth Mover’s Distance (EMD) loss, which is described as follows:

(9) $$EMD={\left(\frac{1}{M}\sum_{k=1}^{M}{\left|CDF_{y}\left(k\right)-CDF_{\hat{y}}\left(k\right)\right|}^{2}\right)}^{1/2}$$

where ${\mathrm{CDF}}_{y}\left(k\right)=\sum_{i=1}^{k}y_{i},\;{\mathrm{CDF}}_{\hat{y}}\left(k\right)=\sum_{i=1}^{k}{\hat{y}}_{i}$ denotes the cumulative distribution function. $y=\left\{y_{1},y_{2},y_{3},\ldots ,y_{M}\right\}$ and ${\hat{y}}_{i}=\;\left\{{\hat{y}}_{1},{\hat{y}}_{2},{\hat{y}}_{3},\ldots ,{\hat{y}}_{M}\right\}$ represent the ground truth and predicted results. *M* denotes the total number of aesthetic score bins.

After obtaining the aesthetic distribution, the binary classification accuracy and aesthetic quality score based on this distribution are calculated.

### 3.2. Attention-Based Attribute-Guided Aesthetic Module (AGAM)

**Figure 3:**
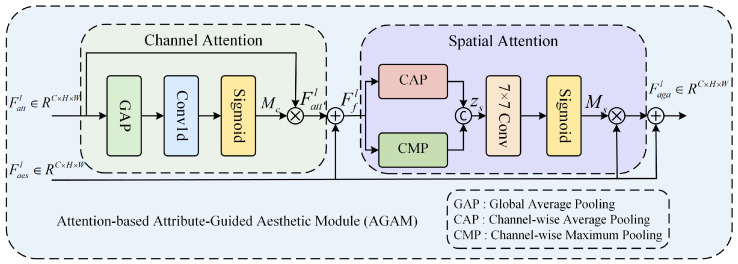
Architecture of the proposed Attention-based Attribute-Guided Aesthetic Module (AGAM).

In order to achieve the guidance of attribute features on aesthetic features, we designed the Attention-based Attribute-Guided Aesthetic Module (AGAM) based on human visual perception characteristics. Neuroscience research shows that when the human brain receives visual stimulation, it prioritizes processing the overall perception of the image, such as light and color. before moving on to a detailed analysis [41,42,43]. Therefore, in AGAM, we utilize channel attention to simulate how the human brain processes the overall perception of the image, and then employ spatial attention to simulate the handling of local detail features, which better calibrates the aesthetic features at both global and local aspects. The structure of AGAM is shown in Figure 3.

In AGAM, we utilize attention mechanisms to simulate human visual processing. The key variables are defined as follows:

- $M_{c}\in R^{C\times 1\times 1}$: Channel attention weights;
- $M_{s}\in R^{1\times H\times W}$: Spatial attention weights;
- $F_{att^{\prime }}$: Feature map obtained by applying a channel attention mechanism to $F_{att}^{l}$;
- $F_{f}^{l}$: Intermediate feature map created by fusing $F_{att^{\prime }}^{l}$ and $F_{aes}^{l}$.

We first perform channel attention operations on the attribute feature $F_{att}^{l}$. Global average pooling (GAP) is applied to $F_{att}^{l}$ to aggregate spatial information, followed by a one-dimensional convolutional layer and a Sigmoid activation function to generate the channel attention weights $M_{c}$. These weights are then used to recalibrate the original attribute feature through element-wise multiplication, producing the enhanced attribute feature $F_{att^{\prime }}^{l}$. Finally, the enhanced attribute feature $F_{att^{\prime }}^{l}$ is fused with the original aesthetic feature $F_{aes}^{l}$ via element-wise summation to obtain the intermediate fusion feature $F_{f}^{l}$. This feature represents the initial integration of the attribute and aesthetic branches. The formula is as follows:

(10) $$M_{c}=\mathrm{Sigmoid}\left(\mathrm{Conv}1D\left(\mathrm{GAP}\left(F_{att}^{l}\right)\right)\right)$$

(11) $$F_{att^{\prime }}^{l}=M_{c}⊙F_{att}^{l}$$

(12) $$F_{f}^{l}=F_{att^{\prime }}^{l}+F_{aes}^{l}$$

where Conv1D denotes the one-dimensional convolutional layer and ⊙ denotes the element-by-element multiplication.

Following the channel attention, spatial attention is applied to the intermediate fusion feature $F_{f}^{l}$ to further enhance the aesthetic features from details by focusing on spatially important regions. First, channel-wise average pooling (CAP) and channel-wise max pooling (CMP) are performed on $F_{f}^{l}$, and the results are concatenated along the channel dimension to form the spatial feature $z_{s}$. This feature is then processed by a 7×7 convolutional layer followed by a Sigmoid activation to generate the spatial attention weights $M_{s}$. The large 7×7 convolution kernel is employed to capture broader spatial context and relationships within the feature map. Finally, the spatial attention map $M_{s}$ is applied to the original aesthetic feature $F_{aes}^{l}$ through element-wise multiplication, and the result is combined with the original $F_{aes}^{l}$ via a residual connection to produce the final output $F_{aga}^{l}$. The formula is as follows:

(13) $$z_{s}=\mathrm{Concat}(\mathrm{CAP}(F_{f}^{l}),\;\mathrm{CMP}(F_{f}^{l}))$$

(14) $$M_{s}=\mathrm{Sigmoid}\left({\mathrm{Conv}}_{7\times 7}\left(z_{s}\right)\right)$$

(15) $$F_{aga}^{l}=F_{aes}^{l}+M_{s}⊙F_{aes}^{l}$$

where CAP(·) denotes the channel-wise average pooling layer and CMP(·) denotes the channel-wise maximum pooling layer.

### 3.3. Adaptive Multi-Dilate Rate Convolution Module (AMDM)

**Figure 4:**
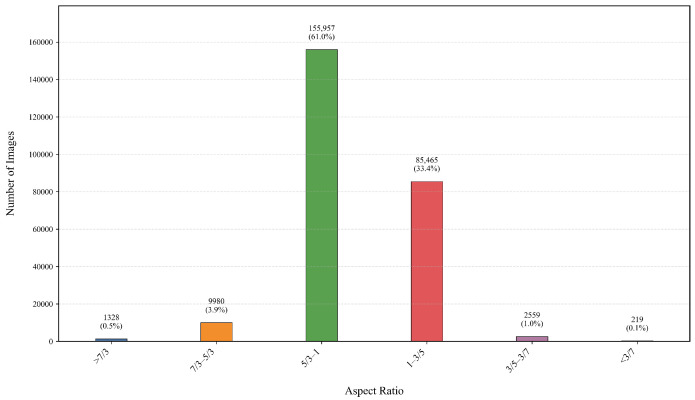
Distribution of image aspect ratios for the AVA dataset.

CNNs typically require fixed-size input images, necessitating the cropping or resizing of original images to achieve scale transformation. However, this preprocessing may disrupt the image’s initial composition due to changes in the aspect ratio. Consequently, it affects the consistency between the cropped input image and the aesthetic label. Therefore, we construct a Multi-Dilated Convolutional Network, consisting of five dilated convolution blocks named Dil Block. The Dil Block is composed of multiple Dil Bottlenecks, with the same number of Dil Bottlenecks in each Dil Block as the Res Bottlenecks in the corresponding Res Block. The Dil Bottleneck is constructed by replacing the 3 × 3 convolution layer in the corresponding Res Bottleneck with our designed Adaptive Multi-Dilate Rate Convolution Module (AMDM). Res Bottleneck and Dil Bottleneck are (a) and (b) in Figure 2, respectively. The module includes parallel convolution kernels with different dilation rates and dynamically combines multiscale features by using an adaptive weighting mechanism, which is adaptive to images of varying aspect ratios. This design largely maintains the aspect ratio of the original image, alleviates the damage of preprocessing, and simulates the adaptive perception characteristic of the human brain on different aspect ratio images. To determine appropriate dilation rates for the dilated convolutions, we analyze the distribution of image aspect ratios in the AVA dataset [44], as shown in Figure 4. It can be seen that more than 99% of the image aspect ratios are distributed in the range of 7:3 to 3:7. Therefore, we selected five dilated convolution kernels corresponding to different aspect ratios, including (1,3), (1,2), (1,1), (2,1) and (3,1), to ensure the applicability of the model to images with different aspect ratios.

The structure of the designed AMDM is shown in Figure 2. The AMDM processes input feature maps using parallel dilated convolutions with adaptive weighting.

- $F_{in}\in R^{C\times H\times W}$: Input feature map;
- $F_{i}$: Feature map from the *i*-th dilated convolution;
- $W=\{W_{1},W_{2},W_{3},W_{4},W_{5}\}$: Adaptive weights for feature fusion;
- $F_{\mathrm{weighted}}$: Weighted concatenation of all dilated convolution outputs;
- $F_{\mathrm{out}}\in R^{C\times H\times W}$: Final output feature map;
- $\Psi_{\mathrm{AMDM}}\left(\cdot \right)$: Nonlinear transformation of AMDM module;
- *K*: Number of Dil Bottlenecks in each Dil Block;
- $B_{j}\left(\cdot \right)$: Transformation of *j*-th Dil Bottleneck.

This process can be described as follows:

First, extract features by 5 sets of parallel dilated convolutions:

(16) $$\begin{array}{c} F_{i}={\mathrm{Conv}}_{3\times 3}^{d_{i}}\left(F_{in}\right),\\ d_{i}\in \{\left(1,3\right),\left(1,2\right),\left(1,1\right),\left.\left(2,1\right),\left(3,1\right)\right\}\\ i=1,2,3,4,5 \end{array}$$

where ${\mathrm{Conv}}_{3\times 3}^{d_{i}}\left(\cdot \right)$ denotes 3×3 dilated convolutions with five different dilation rates.

Then, employ a two-layer convolutional network to obtain *W*, which are used to weight the outputs of different dilated convolutions.

(17) $$W=\;\mathrm{Softmax}\left(\mathrm{GAP}\left({\mathrm{Conv}}_{3\times 3}\left(\mathrm{BN}\left({\mathrm{Conv}}_{3\times 3}\left(F_{in}\right)\right)\right)\right)\right)$$

where BN(·) denotes batch normalization.

Finally, we multiply the corresponding $W_{i}$ and $F_{i}$ and concatenate them to obtain $F_{\mathrm{weighted}}$. Then, we apply a 1 × 1 convolution layer, batch normalization layer, and ReLu activation function to obtain $F_{out}$, which is computed as follows:

(18) $$F_{\mathrm{weighted}}=\mathrm{Concat}\left(W_{i}{*F}_{i}\right),\;i=1,2,3,4,5$$

(19) $$F_{out}=\mathrm{ReLu}\left(\mathrm{BN}\left({\mathrm{Conv}}_{1\times 1}\left(F_{\mathrm{weighted}}\right)\right)\right)$$

The interaction between AGAM and AMDM follows a hierarchical architecture where these modules do not directly communicate, but rather interact through the network’s layered structure. The output from the AGAM module is fed into the Dil Block, which consists of a series of Dil Bottlenecks. Within each Dil Bottleneck, the AMDM module operates as a core component. This hierarchical interaction can be mathematically represented as follows:

(20) $$F_{\mathrm{aga}}^{l}=\Psi_{\mathrm{AGAM}}^{l}\left(F_{\mathrm{att}}^{l},F_{\mathrm{aes}}^{l}\right)$$

(21) $$F_{\mathrm{aes}}^{l+1}=\Psi_{\mathrm{aes}}^{l+1}\left(F_{\mathrm{aga}}^{l}\right)=B_{K}\circ B_{K-1}\circ \ldots \circ B_{1}\left(F_{\mathrm{aga}}^{l}\right)$$

where ∘ denotes function composition.

The process of obtaining the output *Y* from the input *X* through the Dil Bottleneck $B_{j}\left(X\right)$ is defined as follows:

(22) $$Y=\mathrm{ReLU}\left(\mathrm{BN}\left({\mathrm{Conv}}_{1\times 1}\left(\Psi_{\mathrm{AMDM}}\left(\mathrm{ReLU}\left(\mathrm{BN}\left({\mathrm{Conv}}_{1\times 1}\left(X\right)\right)\right)\right)\right)\right)+X\right)$$

The AMDM module operates within each Dil Bottleneck $B_{j}$, processing features through adaptive multi-dilated convolution while maintaining the original aspect ratio information. This hierarchical design ensures that attribute-guided features from AGAM are progressively refined through multiple AMDM-enhanced transformations before being passed to the next AGAM module.

## 4. Experiments

### 4.1. Databases

To evaluate the performance of the proposed MAADN, we conduct experiments on three commonly used IAA databases, including AVA [44], AADB [40], and PARA [45].

**Figure 5:**
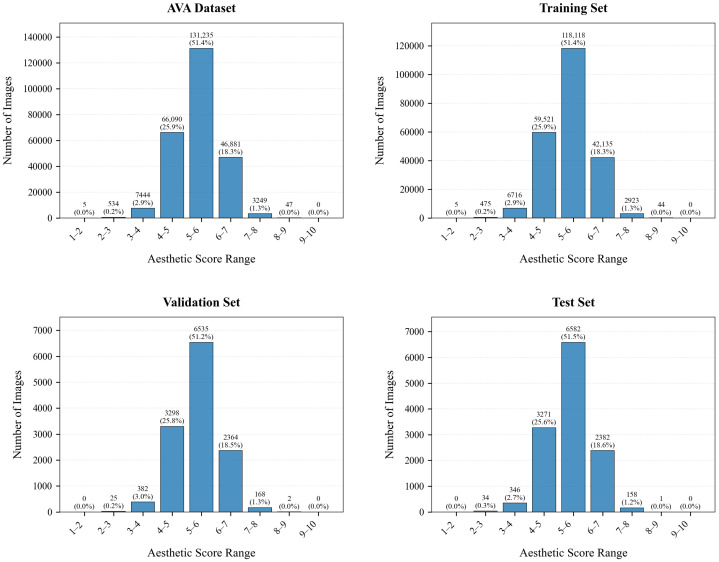
Score histograms for the training, validation, and test splits of the AVA dataset.

AVA Database [44]: contains more than 250,000 images collected from the DPChallenge website, and is currently the largest database for IAA. AVA contains three types of annotations, including aesthetic score distribution (with the range [1, 10]), semantic content, and photographic style. After removing corrupted images, we obtain a final dataset of 229,937 images for training, with 12,774 images each allocated for validation and testing. The comparisons of the score histograms for these splits are shown in Figure 5. It visually demonstrates the high consistency in score distributions across the splits, confirming that our data selection is reasonable.

AADB Database [40]: contains 10,000 images. It not only provides an overall aesthetic score (with the range [1, 5]) for each image but also provides 11 different aesthetic attribute scores. In our experiments, aesthetic attributes were used for pre-training the Attribute Branch. During the aesthetic quality prediction process, we used 8500 images for model training, 500 images for validation, and the remaining 1000 images for testing.

PARA Database [45]: contains 31,220 images. Its annotations mainly consist of aesthetic scores (with the range [1, 5]), aesthetic attribute scores, emotion categories, and scene categories. Aesthetic attributes include layout, shallow depth of field, color harmony, content interest, and lighting. It also provides eight types of emotion category tags and ten types of scene category tags. In this paper, we use 28,220 images for training and the remaining 3000 images for testing the performance of the model.

### 4.2. Implementation Details

In our implementation, we use PyTorch 1.12 to build the proposed MAADN. During training, we first resize the image to 256 × 256 × 3 and then randomly crop it to 224 × 224 × 3 for input and utilize horizontal flipping to enhance the data. In the testing phase, we directly resize the original image to 224 × 224 × 3. The training process consists of three stages. First, the Attribute Branch is pre-trained on the AADB dataset. Then, its parameters are frozen. Finally, the entire MAADN is trained on the target dataset. We use the Adam optimizer for optimization with an initial learning rate of 2 × 10^−5^, a decay rate of 0.1 per 10 epochs, and a batch size set to 32. All experiments are performed on 2 × NVIDIA GeForce RTX 3070 Ti 8G GPUs.

We evaluate the performance of our MAADN from three aspects: binary classification, aesthetic score regression, and aesthetic distribution prediction. For the binary classification task, we evaluate the performance of the model using the accuracy (ACC), which is calculated as follows:

(23) $$ACC=\frac{TP+TN}{TP+TN\;+FP+FN}$$

where TP represents the number of correctly predicted positive samples, TN represents the number of correctly predicted negative samples, FP represents the number of samples incorrectly predicted as positive, and FN represents the number of samples incorrectly predicted as negative.

For the aesthetic regression task, we use the Pearson linear correlation coefficient (PLCC) to evaluate the accuracy of the prediction results, and the Spearman rank order correlation coefficient (SRCC) to measure the prediction monotonicity, respectively, which are computed with the following respective formulas:

(24) $$PLCC=\frac{\sum_{i=1}^{N}\left(Y_{i}-\bar{Y}\right)\left({\hat{Y}}_{i}-\bar{\hat{Y}}\right)}{\sqrt{\sum_{i=1}^{N}{\left(Y_{i}-\bar{Y}\right)}^{2}\sum_{i=1}^{N}{\left({\hat{Y}}_{i}-\bar{\hat{Y}}\right)}^{2}}}$$

(25) $$SRCC=1-\frac{6\sum_{i=1}^{N}{\left(v_{i}-p_{i}\right)}^{2}}{N\left(N^{2}-1\right)}$$

where *N* denotes the number of test images, $Y_{i}$ and ${\hat{Y}}_{i}$ denote the labeled value and score prediction of the ith image, and $\bar{Y}$ and $\bar{\hat{Y}}$ denote the mean of all $Y_{i}$ and ${\hat{Y}}_{i}$, $v_{i}$ and $p_{i}$ denote the true ranking position and predicted ranking position of the ith image.

For the aesthetic distribution prediction task, we use Earth Mover’s Distance (EMD) to evaluate the performance of the model. The EMD formula is shown in Equation (9).

### 4.3. Performance Evaluation

We evaluate the performance of the proposed MAADN on three datasets: AVA, AADB, and PARA. On the AVA dataset, we evaluate the performance of our MAADN in aesthetic binary classification (ACC), aesthetic score regression (SRCC and PLCC), and aesthetic distribution prediction (EMD). As for the AADB and PARA datasets, the evaluation is conducted using SRCC, PLCC, and ACC. To calculate the ACC, we set a classification threshold for aesthetic scores. Images with total aesthetic scores above the threshold are considered to have high aesthetic quality, while other images are considered to have low aesthetic quality. This threshold is 5 on the AVA dataset and 3 on the AADB and PARA datasets. The experimental results are summarized in Table 1, Table 2, and Table 3, respectively. The best and second-best results are marked with bold and underlined, while a “-” indicates that the result is not available.

From Table 1, we can observe that our MAADN achieves the best results in both predicting monotonicity (SRCC) and accuracy (PLCC), achieves the second-best performance in terms of distribution similarity (EMD), but is slightly lacking in terms of classification accuracy (ACC). Our MAADN employs a multi-level attribute-guided aesthetic mechanism and AGAM to capture aesthetic features from global to local. This hierarchical feature extraction mechanism simulates the cognitive process of the human visual system from the global to the local level, allowing it to focus on learning continuous quality scores rather than hard threshold division. We believe that accurately predicting rankings, scores, and distributions is more critical than binary judgments. This indicates the overall advantages of our MAADN. For AFDC [22], which also uses dilated convolutions, our MAADN achieves better results in SRCC, PLCC, and EMD. This is because we propose the AMDM module, which can adaptively weight the features extracted by different dilated convolution kernels, enabling the network to better alleviate the negative impact of image preprocessing. For MUSIQ [46], it employs a vision transformer architecture with high computational complexity, achieving favorable results only on the large-scale AVA dataset, while performing poorly on the AADB and PARA datasets. As can be seen from Table 2 and Table 3, our MAADN achieved the best SRCC and PLCC on both the AADB and PARA datasets, demonstrating the broad adaptability of our MAADN.

**Table 1:** Performance comparison of different methods on AVA dataset.

Method	SRCC ↑	PLCC ↑	ACC ↑	EMD ↓
A-Lamp(VGG16) [47]	-	-	82.50%	-
NIMA(VGG16) [12]	0.592	0.610	80.60%	0.052
NIMA(Inception) [12]	0.612	0.636	81.51%	0.050
GRF-CNN(VGG16) [48]	0.676	0.687	80.70%	0.046
GRF-CNN(Inception) [48]	0.690	0.704	81.81%	0.045
AFDC(ResNet50) [22]	0.649	0.671	83.24%	0.045
MUSIQ(VIT) [46]	**0.726**	**0.738**	81.50%	-
HLA-GCN(ResNet101) [49]	0.665	0.687	**84.60%**	0.043
TAAN(Swim-T) [50]	-	-	76.82%	-
IAFormer(VIT) [31]	0.664	0.674	82.00%	0.065
HNEF(ResNet50) [51]	0.679	0.694	83.90%	**0.040**
SPTF-CNN(VIT) [52]	0.687	0.709	84.50%	0.043
ANKE(EfficientNet) [53]	0.710	0.719	-	0.044
Zhang(ResNet50) [54]	0.664	0.674	82.00%	0.065
CompoNet(ResNet34) [55]	0.678	0.680	83.80%	0.061
MMANet(MobileNet) [56]	0.700	0.715	81.86%	0.048
CILNet(ResNet18) [57]	0.693	0.702	84.20%	0.059
WMPR-Net(ResNet-50) [58]	0.703	0.713	80.20%	0.045
MAADN (ours)	0.714	0.728	81.94%	0.043

The best and second-best results are marked with bold and underlined, while a “-” indicates that the result is not available. The upward arrow (↑) indicates that higher values are better, while the downward arrow (↓) indicates that lower values are better.

**Table 2:** Performance comparison of different methods on AADB dataset.

Method	SRCC ↑	PLCC ↑	ACC ↑
RegNet(AlexNet) [40]	0.678	-	-
PA IAA(DenseNet) [59]	0.715	0.730	70.63%
NIMA(ResNet50) [12]	0.708	0.711	**80.10%**
MLSP(Inception) [60]	0.719	0.717	77.20%
MUSIQ(VIT) [46]	0.683	0.702	75.25%
MMANet(MobileNet) [56]	0.731	0.735	77.36%
WMPR-Net(ResNet-50) [58]	0.719	0.713	-
MAADN (ours)	**0.733**	**0.737**	77.48%

The best and second-best results are marked with bold and underlined, while a “-” indicates that the result is not available. The upward arrow (↑) indicates that higher values are better.

**Table 3:** Performance comparison of different methods on PARA dataset.

Method	SRCC ↑	PLCC ↑	ACC ↑
PA IAA(DenseNet) [59]	0.877	0.919	87.50%
NIMA(ResNet50) [12]	0.891	0.913	**88.60%**
MLSP(Inception) [60]	0.832	0.897	83.70%
MUSIQ(VIT) [46]	0.875	0.918	88.30%
MMANet(MobileNet) [56]	0.895	0.924	87.86%
MAADN (ours)	**0.898**	**0.925**	86.57%

The best and second-best results are marked with bold and underlined. The upward arrow (↑) indicates that higher values are better.

### 4.4. Ablation Study

We conducted some ablation experiments to verify the effectiveness of the proposed MAADN, as shown in Table 4.

Effectiveness of Image Attribute Hierarchy Guidance: We first only use ResNet50 to extract image aesthetic features to directly predict aesthetic quality as the baseline named ’Baseline’ without guidance based on attribute features. Subsequently, we construct a model that achieved the guidance only at the last layer and a model that used multi-level attribute features to guide the aesthetic features, which achieve guidance by adding the corresponding layer attribute features and aesthetic features. These two models are called ’Single-Level Guide’ and ’Multi-Level Guide’ in Table 4, respectively. All constructed models are trained and tested under the same conditions. The experimental results demonstrate that the ’Multi-Level Guide’ model outperforms the ’Single-Level Guide’ model in SRCC, PLCC, and ACC because it better simulates the hierarchical structure of the human visual system and has an important role in the aesthetic evaluation of images.

Effectiveness of Attention-based Attribute-Guided Aesthetic Module (AGAM): To evaluate the effectiveness of the Attention-based Attribute-Guided Aesthetic Module (AGAM), we first construct two comparative variants: ’SG + AGAM’ by replacing the addition operation in the Single-Level Guide model with our AGAM module, and ’MG + AGAM’ by similarly integrating AGAM into the Multi-Level Guide framework. AGAM simulates the brain’s global-to-local visual processing mechanism, selectively enhancing interactions between attribute features and aesthetic features through attention mechanisms. Experimental results in Table 4 confirm that AGAM improves performance in both aesthetic binary classification and score regression tasks, demonstrating its capability to enhance attribute-aesthetic feature interactions and thereby strengthen the attribute guidance process. It is noteworthy that the performance gain observed under the multi-level guidance structure is more pronounced than that under the single-level guidance. This demonstrates a synergistic effect between AGAM and the hierarchical guidance structure.

Effectiveness of Adaptive Multi-Dilate Rate Convolution Module (AMDM): We construct the ‘Baseline + AMDM’ by replacing the standard 3 × 3 convolution in the baseline model, the `SG + AMDM’ and ‘MG + AMDM’ variants by replacing the 3 × 3 convolution layer in the Res Bottleneck of the Aesthetic Branch within the Single-Level Guide and Multi-Level Guide models, respectively. Experimental results in Table 4 demonstrate consistent performance improvements across all these configurations, confirming that AMDM effectively mitigates image quality degradation caused by preprocessing through its adaptive weighting of dilated convolution kernels with different dilation rates. The incorporation of the AMDM module brings stable and relatively consistent performance improvements in every configuration it is applied to, from the baseline to both single-level and multi-level guidance models. This robust and generalizable effectiveness stems from its core function: adaptively fusing multi-scale features to preserve the original image composition, thereby mitigating the information loss typically caused by standard image preprocessing.

Synergistic Integration: The intermediate model `SG + AGAM + AMDM’, which incorporates both proposed modules into the single-level guidance framework, already demonstrates superior performance compared to models using either module alone. This observation validates the complementary nature of our designs and provides strong justification for their full integration in MAADN. The complete MAADN model, which comprehensively integrates multi-level guidance, AGAM, and AMDM, achieves the highest performance. The results confirm that these components work in concert, with the hierarchical attribute features being effectively refined by AGAM, while the aesthetic branch benefits from the composition-preserving capabilities of AMDM.

### 4.5. Sensitivity Analysis for Hierarchical Selection

A core contribution of our work is the multi-level attribute guidance mechanism. To empirically validate the necessity of our full hierarchical design and to understand the contribution of each level, we conduct a sensitivity analysis in two sequential phases. The first phase evaluates the efficacy of guidance from individual levels, while the second phase investigates the synergistic effects of combining them.

We begin by constructing and evaluating four models to isolate the effect of guidance from individual network levels. These models are designed to be guided exclusively by low-level features (Level 1), by middle-low-level features (Level 2), by middle-high-level features (Level 3), and by high-level features (Level 4), respectively. The model using high-level feature guidance (Level 4) is identical to ‘SG+AGAM’ in Table 4. The performance of these models on the AVA dataset is presented in the top section of Table 5. As shown in Table 5, guidance from high-level features alone (Level 4) yields the best performance among all single-level configurations. This is intuitive as high-level features capture semantic and compositional attributes that are most directly correlated with global aesthetic judgment.

Building upon the finding that high-level guidance is the most potent, we proceed to investigate whether integrating it with guidance from lower levels could yield a synergistic performance gain. We systematically design models that combine high-level guidance with features from progressively lower levels. This includes a model integrating high and mid-high levels (Levels 3–4), another integrating middle-level guidance (Levels 2–4), and finally, our full model that incorporates guidance from all levels, including the lowest (Levels 1–4). The results are shown in the bottom section of Table 5. Crucially, the model’s performance improves progressively as we incorporate guidance from more levels, with our full model (Levels 1–4) achieving the best results. This demonstrates that while high-level guidance is the most powerful single component, low-level and middle-level features provide complementary information that the network can synergistically integrate with high-level semantics for a more comprehensive aesthetic assessment. This empirically validates the necessity and optimality of our proposed multi-level design.

### 4.6. Statistical Significance Analysis

To rigorously validate the performance improvements of our proposed MAADN, we conduct statistical significance tests comparing it against the baseline model using the Wilcoxon signed-rank test [61]. We perform 10 independent runs with different random seeds on the AVA dataset and record the SRCC, PLCC, and ACC metrics for each run.

The null hypothesis ($H_{0}$) states that there is no significant performance difference between MAADN and the baseline, while the alternative hypothesis ($H_{1}$) states that a significant difference exists. A p-value less than 0.05 indicates statistical significance at the 95% confidence level.

As shown in Table 6, all performance metrics show statistically significant improvements with p-values well below the 0.05 threshold. The baseline results (0.671 SRCC, 0.683 PLCC, 79.80% ACC) align with our previously reported values in Table 1, while MAADN achieves consistent improvements across all metrics (0.714 SRCC, 0.728 PLCC, 81.94% ACC) with smaller standard deviations, indicating better stability.

These results provide strong statistical evidence that the performance gains of MAADN are not due to random chance but represent genuine improvements in aesthetic assessment capability. The low p-values, particularly for SRCC (p=0.007) and PLCC (p=0.005), confirm the robustness of our method’s superiority in both ranking correlation and prediction accuracy.

### 4.7. Computational Efficiency Analysis

To assess the model’s deployment feasibility and quantify the cost of each component, we analyze its parameter count and computational complexity. The results for each key configuration of our model are presented in Table 7. The naming conventions in this table are consistent with those defined in Table 4. This analysis allows for a transparent understanding of the resource overhead introduced by our proposed modules.

As illustrated in the Table 7, the progression from the Baseline to the full MAADN model shows a corresponding increase in complexity. The transition from the single-branch Baseline to the dual-branch Multi-Level Guide doubles the total parameters due to the introduction of the parallel attribute branch. However, because the attribute branch is pre-trained and frozen, the number of trainable parameters remains almost unchanged. This design choice efficiently leverages attribute knowledge without significantly increasing the training cost. Integrating the AGAM into the MG adds a negligible number of parameters. The minimal cost of this module, coupled with the consistent performance gain observed in our ablation study, confirms its high efficiency in enhancing feature interactions through its channel and spatial attention mechanisms. Incorporating the AMDM results in the most significant increase in model complexity, adding approximately 25.47M parameters and 4.03 GFLOPs. This substantial cost is attributed to the module’s core design, which replaces standard convolutions with multiple parallel dilated convolutions and an adaptive weighting network. The ablation experiments demonstrated the validity of this investment, as AMDM proved crucial for preserving the original image composition and mitigating preprocessing damage. The complete MAADN model, which integrates all proposed components, has the largest computational footprint but also delivers the best performance. The analysis clearly shows that this cost is primarily driven by the powerful, yet parameter-heavy, AMDM module. This granular breakdown provides clear guidance for practical deployment. The model’s design offers flexibility: in scenarios with limited computational resources or where input images have standard aspect ratios, one could opt for a simplified variant, such as MG + AGAM, which removes the AMDM module while retaining the benefits of multi-level attribute guidance and attention-based feature enhancement. This demonstrates the adaptable nature of our architectural contributions. A comparison with standard Vision Transformer architectures indicates that the computational complexity of our full MAADN model is on par with the ViT-B/16 [62] model, yet it is considerably more efficient than the much larger ViT-L/16 [62] model, demonstrating a favorable balance between performance and computational cost.

### 4.8. Visualization Experiment

**Figure 6:**
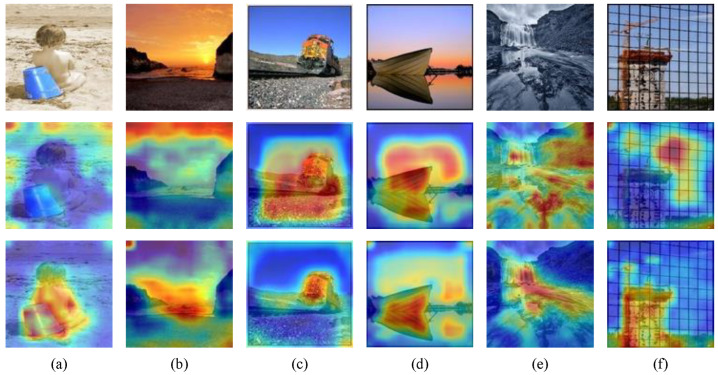
Grad-CAM visualizations of model predictions for six sample images (**a**–**f**), with the first row displaying the original images, the second row showing the predictions from ResNet50, and the third row presenting the predictions from the proposed MAADN. The red is the highest attention area, yellow is next, green again, and blue is the lowest attention color.

In order to intuitively demonstrate the explainability of the proposed MAADN model, we use Grad-CAM to visualize the regions of interest across six images from the AVA, AADB, and PARA datasets, as shown in Figure 6. The first row displays the original images, the second row shows the Grad-CAM visualizations from the ’Baseline’ model, which uses ResNet50 to directly extract image features without our proposed multi-level guidance from attribute to aesthetic, AGAM, and AMDM, and the third row presents the Grad-CAM visualizations from our MAADN model. The red background is the highest attention area, yellow is next, green again, and blue is the lowest attention color.

As illustrated in Figure 6, the attention regions of our MAADN exhibit a significantly higher degree of alignment with human perceptual focus compared to the baseline. This improvement can be attributed to our design, which effectively simulates the hierarchical and adaptive nature of the human visual system. Specifically, the AGAM module guides the model to prioritize overall perceptual attributes before refining details, while the AMDM module helps preserve the original composition, reducing distortion from preprocessing. The specific analysis is as follows: For Figure 6a,b, where the overall color palette is relatively uniform, the baseline model exhibits scattered attention. In contrast, our model accurately concentrates on the main subjects and areas with distinct color variations, such as the bucket in Figure 6a. This indicates that our model has effectively learned to prioritize attributes like “Vivid Color” and “Object” emphasis. Furthermore, since Figure 6a,b represent a portrait and a landscape, respectively, these results confirm our model’s robust performance across different scene types. As shown in Figure 6c,d, our model excels in understanding compositional rules. It precisely locates the main subject at a position approximating the “Rule of Thirds” in Figure 6c, and its attention map in Figure 6d exhibits a strong symmetrical pattern that mirrors the image’s prominent “Symmetry”. This demonstrates a successful learning of key compositional attributes. Finally, we include and analyze challenging cases in our visualization experiments to directly probe the model’s boundary conditions and applicability. Figure 6e,f showcase our model’s capability under challenging conditions. For the monochrome image in Figure 6e, which lacks the crucial attribute of Color, MAADN still generates an attention map highly consistent with human perception. In the severely occluded scene in Figure 6f, our model successfully focuses on the primary subject behind the obstruction, demonstrating robust performance in complex scenarios.

In summary, these visualizations confirm that MAADN robustly leverages aesthetic attributes for precise focus localization. It performs effectively not only in general scenes but also under demanding conditions involving missing attributes or complex layouts, highlighting its strong generalization capability and explainability. Simultaneously, these visualization results provide strong qualitative evidence that the aesthetic concepts (e.g., color, symmetry) learned by our attribute branch pre-trained on AADB have successfully transferred to the AVA and PARA datasets, thereby validating their universal applicability.

## 5. Conclusions

In this paper, we propose a Multi-level Attribute-Guided-based Adaptive Multi-Dilated Convolutional Network (MAADN) for image aesthetic evaluation. Our method introduces a hierarchical guidance mechanism from attribute features for the IAA task. Specifically, we propose an Attention-based Attribute-Guided Aesthetic Module (AGAM) that enhances the guidance effect using visual attention mechanisms. We also design an Adaptive Multi-Dilated Rate Convolution Module (AMDM), which reduces the impact of the image preprocessing process on image aesthetics by employing convolution kernels with different dilation rates in parallel to simulate the adaptive perception characteristics of the human brain for images with different aspect ratios. Extensive experiments demonstrate the superior performance of MAADN. However, the proposed approach has certain limitations. The parallel multi-dilation rate convolution kernels in the AMDM introduce more parameters, increasing the computational burden of the model. Furthermore, the AGAM module may exhibit limitations when processing images with unreliable attribute representations or significant domain shifts from the photographic styles seen during training. In future work, we will explore more efficient architectural designs or optimization strategies to reduce computational complexity and enhance the model’s robustness across diverse aesthetic domains.

## Figures and Tables

**Table 4:** Ablation study on AVA dataset.

Method	SRCC ↑	PLCC ↑	ACC ↑
Baseline	0.671	0.683	79.80%
Baseline + AMDM	0.685	0.694	80.43%
Single-Level Guide	0.689	0.701	80.76%
SG + AGAM	0.692	0.703	80.85%
SG + AMDM	0.701	0.710	81.39%
SG + AGAM + AMDM	0.704	0.716	81.64%
Multi-Level Guide	0.696	0.706	81.05%
MG + AGAM	0.701	0.710	81.39%
MG + AMDM	0.709	0.719	81.78%
MAADN (ours)	**0.714**	**0.728**	**81.94%**

The best results are marked with bold. The upward arrow (↑) indicates that higher values are better.

**Table 5:** Performance comparison of different hierarchical guidance strategies on the AVA dataset.

Guidance Hierarchy	SRCC ↑	PLCC ↑	ACC ↑
Level 1	0.681	0.703	80.19%
Level 2	0.688	0.698	80.61%
Level 3	0.689	0.700	80.68%
Level 4	0.692	0.703	80.85%
Levels 3–4	0.697	0.706	80.99%
Levels 2–4	0.698	0.708	81.13%
Levels 1–4 (ours)	**0.701**	**0.710**	**81.39%**

The best results are marked with bold. The upward arrow (↑) indicates that higher values are better.

**Table 6:** Statistical significance analysis between baseline and proposed MAADN on AVA dataset.

Metric	Baseline	Proposed (MAADN)	Wilcoxon *p*-Value
SRCC	0.671±0.008	0.714±0.006	0.007 *
PLCC	0.683±0.007	0.728±0.005	0.005 *
ACC (%)	79.80±0.35	81.94±0.28	0.013 *

* indicates statistical significance (*p*-value < 0.05).

**Table 7:** Analysis of model complexity.

Model Configuration	Total Params (M)	Trainable Params (M)	GFLOPs
Baseline	25.58	25.58	4.13
Multi-Level Guide	51.16	25.58	8.26
MG + AGAM	51.17	25.59	8.28
MG + AMDM	76.64	51.06	12.31
MAADN (ours)	76.65	51.07	12.33
ViT-B/16 [62]	85.81	85.81	11.29
ViT-L/16 [62]	303.31	303.31	39.86

## Data Availability

The original data presented in the study are openly available in the AVA dataset [44] (available at https://github.com/imfing/ava_downloader, accessed on 17 November 2025), the AADB dataset [40] (available at https://huggingface.co/datasets/Iceclear/AADB, accessed on 17 November 2025), and the PARA dataset [45] (available at https://web.xidian.edu.cn/ldli/en/dataset.html, accessed on 17 November 2025).
